# Infected with Dengue Seven Times, and the Motivation to Study a Science Degree

**DOI:** 10.4269/ajtmh.25-0535

**Published:** 2025-10-23

**Authors:** Sarita Doménica Bautista-Arcentales, Ángel Sebastián Rodríguez-Pazmiño

**Affiliations:** One Health Research Group, Universidad de Las Américas, Quito, Ecuador

One day, when I was eleven years old, I woke up with a high fever and such intense muscle pain that I could not even get out of bed. Worried, my parents quickly took me to the Colón Clinic in Esmeraldas. I remember sitting nervously in the waiting room while they reassured me not to worry. After a blood sample was taken for a serological test, the doctor returned and calmly told us, “It’s dengue.” I felt scared and confused. I did not fully understand what it meant, but I knew my daily routine was about to change.

What at first seemed like an isolated episode eventually became routine.

Over the years, I have had dengue seven times. The symptoms no longer surprised me, and by the seventh infection, I had accepted it with resignation, thinking, “the same thing again.”

I was born and raised in Atacames, a coastal town in the province of Esmeraldas, near the beach. I lived there until 2022, when I moved to Quito, in the Ecuadorian highlands, to attend university. In Atacames, dengue fever never completely disappears. The *Aedes aegypti* mosquito finds a perfect habitat in the local conditions: a poor sewer system, rains that flood streets and yards, and houses built with precarious materials that allow leaks and water accumulation. Although I acknowledge that my family does not live in vulnerable conditions, I am aware that many of my neighbors face these realities on a daily basis. Neighborhoods such as “La Trece,” with fragile housing and no stable access to drinking water, become settings where poverty fuels the spread of the disease.

The figures from the country’s health institutions confirm what I have experienced. Esmeraldas ranks in the middle in terms of dengue prevalence within the country, being above provinces such as Santa Elena, but well below the critical levels of Guayas or Manabí. However, behind the numbers are the stories of friends hospitalized, schoolmates absent due to fever, and entire families living in fear that a typical case will develop into its hemorrhagic form.

Over the years, I reflected on what could be changed to reduce dengue in my city. For me, the most urgent issue is to improve sewage and drainage in the poorest neighborhoods, along with community education campaigns that teach people how to eliminate breeding sites and identify warning signs. My vision encompasses both structural and social aspects, including adequate infrastructure, citizen participation, and rapid access to health services.

During my time at school, each episode meant prolonged absences of up to two weeks, during which I was confined to bed, missing classes, projects, and special activities while my family cared for me. My teachers were supportive, allowing me to make up missed work, though I often felt frustrated watching my classmates carry on without me. At home, the illness imposed a routine of shared care and constant concern, especially from my mother and grandfather, who made sure I stayed hydrated, rested, and took only acetaminophen. Thanks to my family’s dedication, I never developed hemorrhagic dengue.

One of the experiences that affected me most occurred in 2024, when I was living in Quito for my university studies. I had just returned from spending my end-of-semester vacation in Atacames, as I always did, when, a week before classes began, I started to feel the all-too-familiar symptoms of dengue. I went to a doctor, but at the time, no rapid tests for dengue were available. He ordered a blood test, and although my platelet count was slightly low, the results were reported as normal. Based on that, the doctor dismissed the possibility of dengue.

Instead, he prescribed antibiotics and ibuprofen—medications I knew were inappropriate. Antibiotics would be ineffective, and ibuprofen could exacerbate the condition. Since I recognized the symptoms from previous infections, I chose not to take them. I told my mother what was happening, and eventually, I had to travel while sick to Atacames. At the Colón Clinic, my dengue diagnosis was confirmed, and I spent the rest of the illness recovering at home. That experience left a deep impression on me, showing how, while health professionals on the coast are accustomed to diagnosing dengue, in the Sierra, a lack of exposure can lead to clinical errors.

That experience was a turning point. Amid my fever, I wondered how it would be possible to get a quick and accurate result without relying solely on the doctor’s experience. Years later, at university, I heard names like PCR and ELISA for the first time and realized that this was the answer I had imagined as a child. What began as a wish during illness ultimately became a life goal: to harness science and technology to transform uncertainty into certainty and vulnerability into prevention. Living with dengue influenced my decision to study Biotechnology Engineering at the Universidad de Las Américas (UDLA) in Quito, Ecuador. Although I initially pursued this field because of my love for animals, I soon discovered that science could be a powerful tool for improving people’s health.

Since starting university, my experience has been generally positive. At first, living far from home and adapting to a new city, with its different pace, unfamiliar people, and the need to manage life independently, was challenging. Gradually, I found my place. As the semesters pass, I feel more comfortable in my program and increasingly confident in my abilities. At UDLA, I joined the research team led by Miguel Ángel García Bereguiaín, which focuses on zoonotic and infectious diseases, with dengue being a priority. Here, I can work directly with arboviruses and observe qPCR techniques in practice. It is remarkable to me how a single sample can reveal so much about a virus, and how concepts from class suddenly come alive at the bench. Each day in the lab is an opportunity to learn, refine my skills, and explore how biotechnology can be applied to real-world solutions. I feel fortunate to be able to use this stage of my life to grow as a scientist and gain experiences I never imagined possible.

In this research group, I met Sebastián, a doctoral student who grew up in the Sierra, far from the reality of arboviruses. During one of my first PCR practices with him, as we waited for the thermocycler to finish its run, we shared personal stories to break the ice. Then, I told him that one of the most unusual experiences of my life was contracting dengue multiple times. I explained that although dengue was fairly common where I lived, I had never met anyone else who had experienced it as often as I had. For him, hearing that someone had contracted dengue seven times was surprising. He was accustomed to working with samples from the Coastal and Amazon regions, but he had never met anyone who had experienced the disease firsthand.

I feel that this contrast enriched the team. While researchers focus on evaluating molecular diagnostic protocols, I bring a personal perspective that reminds us that behind each sample there is a patient, a family, and a social context. The group’s projects also include validating commercial kits and developing in-house methods to measure sensitivity, specificity, and costs, thereby strengthening epidemiological surveillance and providing accessible tools in vulnerable communities. In this context, my experience serves as an additional motivation to lend each result a human meaning.

On the other hand, my case challenges the notion that dengue is solely a disease of poverty. While statistics show a strong association between poverty and transmission, my story demonstrates that even those who do not live in the most precarious environments are exposed. The simple fact of living in an endemic city or visiting relatives in vulnerable neighborhoods poses a constant risk.

Today, looking back, I recognize that living with dengue so many times left me with lessons learned and fears, but also motivation. What in childhood were days of weakness and absences from school, in adulthood became the decision to seek answers from science. My story is about a student who turned pain into drive and who today works to ensure that future generations have more effective diagnoses and treatments.

For Sebastián and his colleagues, to know this experience was also a reminder that research is not just about protocols and statistics, but about the human lives it affects. The convergence of personal experience and scientific vocation makes these stories testimonies of resilience and hope in the face of a public health problem that continues to impact Ecuador and the entire region.

